# Study on the Mechanical Properties and Mechanism of a Nickel-Iron Slag Cement-Based Composite under the Action of Sodium Sulfate

**DOI:** 10.3390/ma16217041

**Published:** 2023-11-04

**Authors:** Jingyu Zhang, Yuwan Zhou, Sili Chen, Jinzhu Meng, Junxiang Wang

**Affiliations:** 1 School of Materials Science and Engineering, Shenyang University of Technology, Shenyang 110870, China; zjy7019@sut.edu.cn (J.Z.);; 2 School of Architecture and Civil Engineering, Shenyang University of Technology, Shenyang 110870, China; 3 China Railway Guangzhou Engineering Bureau Group Shenzhen Engineering Co., Ltd., Guangzhou 510660, China

**Keywords:** nickel-iron slag cement-based composite, triaxial compression test, mechanical properties, microscopic mechanism

## Abstract

The accumulated amount of nickel–iron slag has increased with the rapid development of the nickel-iron industry. To determine a method for comprehensively utilizing nickel–iron slag, triaxial compression tests of nickel–iron slag cement-based composite materials under the action of sodium sulfate were conducted, and the effects of the sodium sulfate concentration on the stress-strain relation, shear strength, cohesion, and internal friction angle of the composite materials were analyzed. In addition, the influence mechanism of the nickel–iron slag content and sodium sulfate concentration on the composite was examined. The results revealed that the stress–strain curve of the nickel–iron slag cement-based composites reflected softening. With the increase in the sodium sulfate concentration, the brittleness increased, while the shear strength, cohesion, and internal friction angle decreased; the addition of nickel–iron slag slowed down the rate at which these parameters decrease. Scanning electron microscopy images revealed that nickel–iron slag can improve the internal structure of the cement composite soil, enhance its compactness, and improve its corrosion resistance. The optimum nickel–iron slag content of 14% can improve the cementitious composites’ resistance to sodium sulfate erosion in terms of solid waste utilization and cementitious soil performance. The results obtained can provide technical parameters for preparing and designing cement-based composite materials as well as certain theoretical significance and engineering reference value.

## 1. Introduction

With the rapid development of urban industrialization in China, the increasing scale of human engineering and economic activities and infrastructure construction has expanded continuously; hence, soil–cement composites are widely used in roadbed reinforcement, foundation treatment, foundation pit retaining wall development, and antiseepage construction projects [1,2,3]. Owing to the low winter temperatures in northeast China, soil–cement composites are prone to frequent frost heave and ablation; furthermore, they can be eroded by groundwater and chemical solutions, resulting in the cracking and soil–cement settlement and other phenomena [4,5,6]. Therefore, finding suitable mineral admixtures to improve the mechanical properties and durability of soil–cement is important.

**Figure 1:**
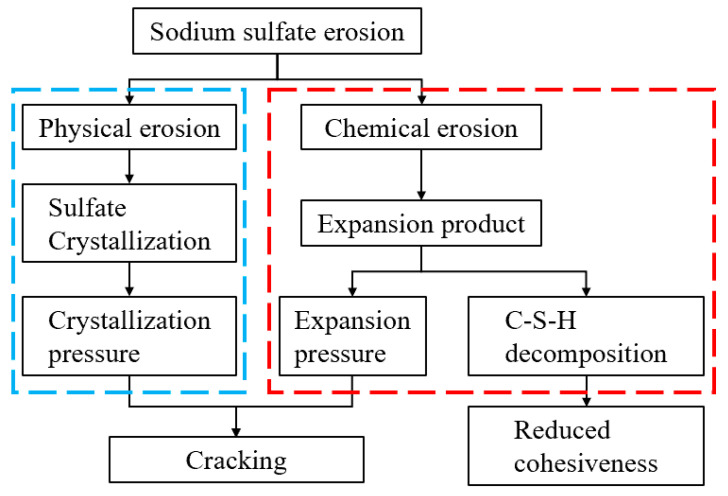
Mechanism of sulfate erosion.

Nickel–iron slag is the granulated solid waste produced by the smelting of laterite nickel ore into a nickel–iron alloy at high temperatures of 1000–1600 °C. Compared with other metallurgical slag, the discharge of nickel–iron slag is large, with a low utilization rate, which has become another major problem of metallurgical slag treatment. Many nickel–iron slag storage facilities sit idle and landfills occupy a large land area, damaging the environment and severely challenging the nickel-iron smelting industry’s sustainable and healthy development. Therefore, there is an urgent need to conduct scientific and systematic research on using nickel–iron slag to enable its large-scale resource utilization in the building materials for the nickel-iron smelting industry. According to statistics, the production of 1 t of nickel generates ~6–16 t of nickel–iron slag, which corresponds to one of the fourth largest industrial waste slags in China [7,8,9,10]. This nickel–iron slag is mainly stored inefficiently in high-density landfills, which wastes a considerable portion of land, threatens the ecological environment of cities, and restricts the development of the nickel–iron industry considerably. Domestic and foreign researchers have studied the comprehensive utilization of nickel–iron slag. Liangyou et al. [11] reported that the activity of the nickel–iron slag powder can be improved by adding activators, such as calcium oxide and sodium sulfate, and the hydration reaction rate of the C–S–H gel system can be accelerated. Yang et al. [12] and Li et al. [13] have reported that the optimal ratio of the nickel–iron slag, activator, and colloidal sand can be determined under a certain static load strength. Bao et al. [14] investigated the freeze resistance and microstructure of concrete by replacing fine aggregates with nickel–iron slag in equal amounts and reported that, at a nickel–iron slag content of 40%, a high amount of the C–S–H gel could be produced, which not only can enhance the compactness of the internal structure but also improve its freeze resistance. Suryaningrat et al. [15] used nickel–iron slag of the same quality instead of cement to examine various properties of mortar and reported that the nickel–iron slag content increased proportionally with the fluidity of the mortar. In the final stages of their test, nickel–iron slag began to react with the mortar, reducing the porosity of the mortar. Dourdounis [16] comparatively analyzed the microstructure of high-alumina cement prepared using nickel–iron slag powder and ordinary cement and reported that high-alumina cement prepared with nickel–iron slag powder exhibited low strength in the early stage; however, its strength improved considerably with the increase in the erosion age, and it also satisfied various performance indexes of cement. Dafang [17] incorporated finely ground nickel–iron slag into cement to study the effect of its fineness (median particle size distribution of 11.65 and 2.89 μm) on hydration activity. The results showed that nickel–iron slag inclusion led to a secondary reaction with it and consumed the calcium hydroxide in the hydration products of cement; additionally, an increase in the nickel–iron slag’s fineness would enhance its participation degree in the reaction. Nickel–iron slag within a certain dosage plays the role of refining the filling of pore sizes of cement. However, the excessive dosages adversely affect the pore structure of hardened slurries. With the development and utilization of China’s coastal resources, many structurally complex and large-scale marine projects have been built near seawater. Our country is in a marine environment, and the buildings often suffer from erosion due to salt solution. The composite erosion and damage by some salt solutions are more severe than others. Hence, the constructed geotechnical structures need to have a long service life, and the short destruction period will cause serious economic losses. Therefore, an in-depth study on the erosion of hydraulic soil in compound salt solutions is vital. Yang [18] and Xing [19] used different aggressive ions to simulate the soluble salt environment for investigating the effect of hydraulic soil erosion by high concentrations of soluble salts. The results of their studies showed that Cl^−^, Mg^2+^, and SO_4_^2−^ would have negatively affected the strength of hydraulic soil. Figure 1 shows the sulfate erosion mechanism reported by Santhanam M et al. [20]. They conducted a categorization study on sulfate erosion and showed that different erosion mecanisms led to different erosion results. Chandra et al. [21] investigated the sulfuric acid resistance of polyferric nickel slag mortar and reported that polyferric nickel slag mortar contained a large amount of the sodium magnesium alumino-silicate hydrate (N–M–A–S–H) gel, which rendered better sulfuric acid corrosion resistance to the mortar. Qi Taishan et al. [22] investigated the effect of nickel–iron slag powder from the blast furnace on the hydration characteristics of cement-based composite cementing materials, and the results revealed that the nickel–iron slag could reduce the hydration heat release rate. Moreover, with the progress of the hydration reaction, the Ca/Si content of the C–S–H gel in the hydration product was lower than that of cement.

The abovementioned studies on nickel–iron slag are mainly applicable to cement and concrete. Some researchers have used nickel–iron slag to prepare cement and new wall materials [23,24,25] as well as for the recovery of valuable metals [26,27], glass-ceramics [28], and inorganic mineral fibers [29,30]. Currently, few studies have reported the corrosion resistance of cementitious composites formed by mixing the nickel–iron slag into silty clay. In this study, the triaxial compression properties of nickel–iron slag cement-based composites under the action of sodium sulfate were investigated, and the microstructure of the cement-based composites was analyzed via scanning electron microscopy (SEM) to reveal the influence mechanism of nickel–iron slag on the corrosion resistance of cement-based composites.

The above research results indicate that comprehensive nickel–iron slag utilization is now garnering extensive attention at home and abroad. Moreover, the feasibility of its application in concrete and the performance impact it brings have been focused on by many scholars. Ruqayah AL-Khafaji et al. [31] reveal that the binary system enhanced the physical and mechanical properties of the soft soil. The optimum binder achieved in this study was 6% (25% GGBS and 75% CKD). However, the current research on nickel–iron slag still lacks systematicity, and understanding of nickel–iron slag production by different ore sources and production methods remains lacking, causing considerable differences between the results of various studies. The research on nickel–iron slag concrete is also mostly at the level of net paste and collodion, and systematic research on concrete remains lacking. Therefore, this project systematically researched the workability, mechanical properties, and long-term durability properties of one type of nickel–iron slags (laterite type) applied to concrete by analyzing, organizing, summarizing, and classifying the properties of different nickel-iron slags. At present, there has been little research on the corrosion resistance of a cementitious composite formed by mixing nickel–iron–slag with silty clay. This paper studied the triaxial compression properties of nickel–iron slag cement-based composites under the action of sodium sulfate. The microstructures of these composites were analyzed using SEM to reveal the mechanism of the influence of nickel–iron slag on the corrosion resistance of cement-based composites. This study comprehensively discusses the feasibility of nickel–iron slag application in concrete, which is conducive to promoting the consumption and utilization of solid waste nickel–iron slag with great economic benefits and social significance.

## 2. Preparation and Testing of Materials

### 2.1. Testing of Raw Materials

#### 2.1.1. Nickel–Iron Slag

**Figure 2:**
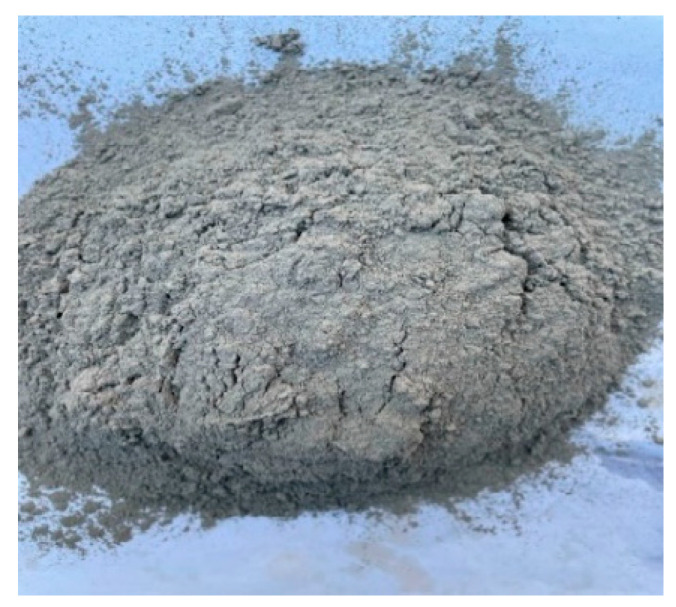
Nickel–iron slag powder.

**Figure 3:**
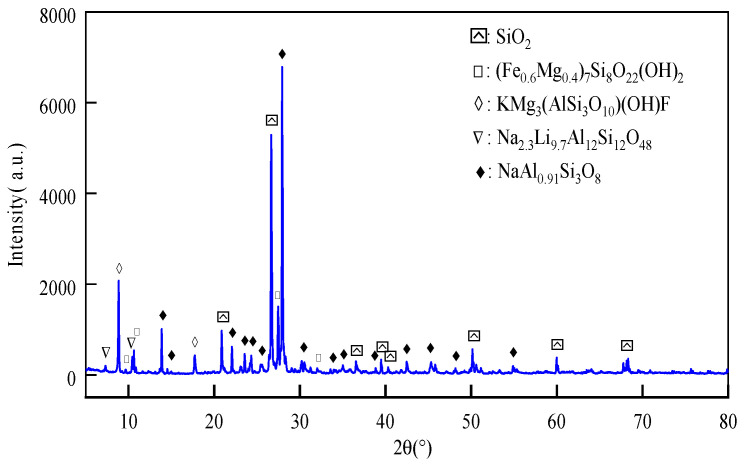
X-ray diffraction (XRD) pattern of the nickel–iron slag.

Nickel-iron slag was provided by a metallurgical material company located in Anshan, Liaoning Province, China. First, the nickel laterite ore was crushed to 50–150 µm; subsequently, we added the reducing agent anthracite and cosolvent limestone. The reduction and smelting output of the nickel content was greater than 10% of the crude ferronickel. Finally, for further refining and enrichment of the nickel content to more than 20%, the converter for producing stainless steel was used to produce refined ferronickel alloy. The nickel–iron slag was calcined at 600 °C, and the calcination time was controlled within 3 h. The calcined nickel–iron slag was ground using a vertical planetary ball mill by Changsha Tianchuang Brand Powder Technology Co. (Changsha, China) for 60 min, with a ball mill speed of 300 r/min, and the polished nickel–iron slag was grayish white (Figure 2). Table 1 shows how X-ray diffraction (XRD, 6.5 version) was employed to analyze the mineral composition of the nickel–iron slag, and determine its content. Table 1 and Figure 3 show the chemical composition and XRD pattern of the nickel–iron slag, respectively.

#### 2.1.2. Silty Clay

Silty clay performance tests were conducted according to standards for test methods of concrete’s physical and mechanical properties (GB/T50081-2019 [32]). The test soil mass was selected from silty clay 3 m below the surface of the foundation pit of a construction site in Shenyang, Liaoning Province, China. First, the natural moisture content of the soil was measured. Then, the air-dried and pulverized soil was passed through a 5 mm screen to measure various physical indexes of the soil, such as air-dried moisture content and the liquid-plastic limit (Table 2).

#### 2.1.3. Cement

Ordinary Portland cement (P.O42.5) was selected in this study; its performance indicators meet the Chinese National Standard “General Portland Cement (GB-175)” [33], as shown in Table 3.

#### 2.1.4. Sodium Sulfate

Anhydrous sodium sulfate produced by a company in Tianjin was selected as the erosion material. Table 4 shows its chemical composition.

### 2.2. Sample Preparation

For the production of specimens, the test material should be mixed proportionally using a cement mortar mixer. According to the provisions stipulated in the specification for the design of the mix proportion of cement soil (based on JGJ/T233-2011 [34]), the original soil mass should be dried under air, crushed through a 5 mm sieve, and sealed and stored.

**Figure 4:**
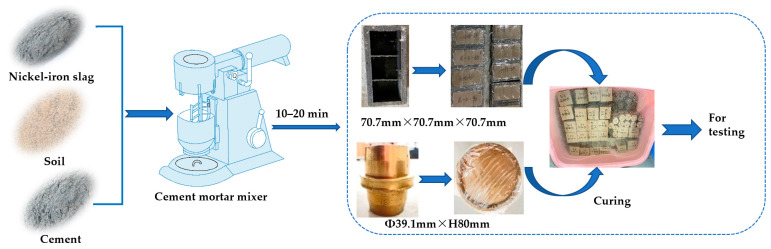
Schematic of the specimen preparation process.

In the nickel–iron slag cement-based composite materials, the optimal mix ratio proposed in a previous study [35] was adopted, i.e., 16% cement and 14% nickel–iron slag with a water–cement ratio of 1.7 (for comparative analysis, 0% nickel–iron slag was prepared simultaneously). Before preparing the sample, the weighed air-dried soil and cement were stirred evenly, the ground nickel–iron slag was added into the mixture successively, and preweighed water was added into the mixture every 3 min. The stirring time was controlled within 10–20 min, and the stirring speed was kept slow at first and then increased to achieve complete and uniform mixing. Then, the evenly mixed cement soil was divided into three parts into the mold, which was evenly coated with petroleum jelly. Then, a compaction hammer was used to vibrate and tamp the mold that holds the soil together. During the compaction process, the compaction hammer must be dropped vertically, the number of compaction events for each layer must be consistent, and the excess material should be scraped off after it is formed. Finally, the specimen must be covered with plastic wrap, and demolding should be conducted after 24 h. After demolding, the specimen surface should be smooth without cracks, faults, and deformation, and the specimen should be marked and placed into a standard room for erosion. The size of the specimen subjected to triaxial compression tests was Φ 39.1 mm × H 80 mm. Figure 4 shows the operation process.

### 2.3. Test Scheme

#### 2.3.1. Erosion Test

A sodium sulfate erosion test was conducted for the specimens prepared from nickel–iron slag cement-based composite materials. The concentration of sodium sulfate was selected according to the requirements of the Code for the Investigation of Geotechnical (GB50021-2001 [36]). After removing the mold, the samples were added into sodium sulfate solutions at different concentrations of 0, 2.5, 6, and 12 g/L for erosion. The erosion time was 90 days, and the triaxial compression test was conducted after the erosion time.

#### 2.3.2. Triaxial Compression Test

The triaxial compression test of the nickel–iron slag cement-based composite material was conducted according to the Chinese Standard JGJ/T 233-2011 [34] (entitled “Specification for mix proportion design of soil–cement”). The size of the specimen was D = 39.1 mm and H = 80 mm. For the test, the sodium sulfate concentrations were 0, 2.5, 6, and 12 g/L, and the confining pressures were 0.3, 0.6, and 0.9 MPa. The TRICK-1 triaxial test measuring and control instrument was used for the triaxial compression test.

#### 2.3.3. Scanning Electron Microscopy

Cement-based composite materials with a cement content of 16%, sodium sulfate concentrations of 0, 2.5, 6, and 12 g/L, 0% nickel–iron slag, and 14% nickel–iron slag were selected as the research objects. Mix the material thoroughly and pour it into the mold. Stabilize for 7 d and unmold the specimen. Enlarge it 20,000 times to analyze the microstructure. After an erosion time of 28 days, SEM images were recorded on a Regulus 8100 system (Hitachi, Tokyo, Japan).

## 3. Test Results and Analysis

### 3.1. Failure Characteristics of the Nickel–Iron Slag Cement–Based Composite Materials

**Figure 5:**
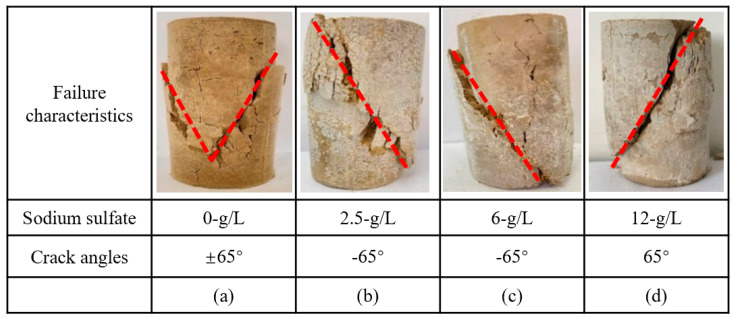
Failure characteristics and crack angles (red lines) of the cement-based composites at a confining pressure of 0.8 MPa. (**a**) Sodium sulfate 0 g/L; (**b**) Sodium sulfate 2.5 g/L; (**c**) Sodium sulfate 6 g/L; (**d**) Sodium sulfate 12 g/L.

The effect of nickel–iron slag on cement-based composites was investigated under the action of 0, 2.5, 6, and 12 g/L of sodium sulfate. Figure 4 and Figure 5 show the failure characteristics of cement-based composites with 14% nickel–iron slag and 0% nickel–iron slag at a confining pressure of 0.8 MPa, respectively.

When the concentration of sodium sulfate is 0 g/L, two main cracks extend from both sides of the soil mass, several cracks appear around it, and the main crack is not connected (Figure 5). Moreover, when the concentration of sodium sulfate is 2.5 g/L, a main crack of ~30° extends from the loading point of the soil top and several cross-penetrating secondary cracks are generated around the main crack. Furthermore, when the concentration of sodium sulfate is 6 g/L, a main crack of ~65° extends from the loading point of the soil top, and the failure form is consistent with that observed when the concentration of sodium sulfate is 2.5 g/L. When the concentration of sodium sulfate is 12 g/L, a main crack of ~90° extends from the soil top at the loading direction, and the damage pattern is consistent with that observed when the concentration of sodium sulfate is 2.5 and 6 g/L.

**Figure 6:**
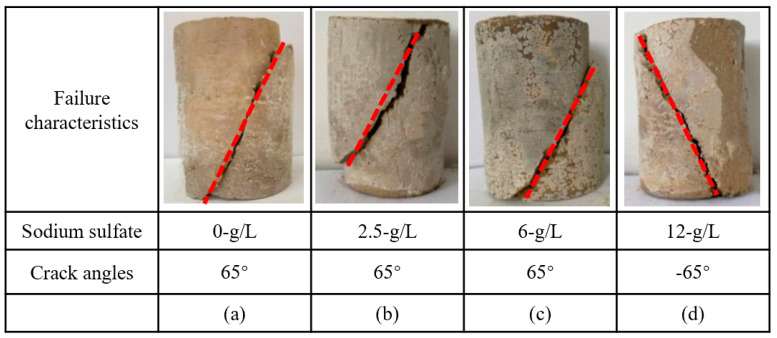
Fracture characteristics and crack angles (red lines) of the nickel–iron slag cement-based composites at a confining pressure of 0.8 MPa. (**a**) Sodium sulfate 0 g/L; (**b**) Sodium sulfate 2.5 g/L; (**c**) Sodium sulfate 6 g/L; (**d**) Sodium sulfate 12 g/L.

When the concentration of sodium sulfate is 0, 2.5, 6, and 12 g/L, a main crack of ~65° extends from the top loading point of the nickel–iron slag cement-based composite material (Figure 6). Furthermore, secondary cracks shown in Figure 4 are not observed around the main crack, and the change characteristics tend to be consistent. The addition of nickel–iron slag is found to inhibit the generation of secondary cracks.

In order to analyze the influence of confining pressure on the failure characteristics of the nickel–iron slag soil–cement composite, the failure characteristics of the nickel–iron slag cement-based composite material at a sodium sulfate concentration of 12 g/L, nickel–iron slag content of 14%, and cement content of 16% were investigated under confining pressures of 0.2, 0.5, and 0.8 MPa.

**Figure 7:**
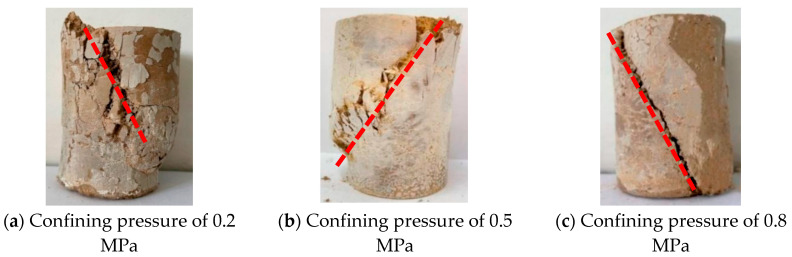
Failure characteristics and crack angles (red lines) of cement-based composite materials with a nickel–iron slag content of 14%.

The confining pressure greatly affects the width, number, and distribution of cracks generated during the failure of the nickel–iron slag cement–based composites (Figure 7). At a confining pressure of 0.2 MPa, several interleaving cracks were observed around the top loading point of the nickel–iron slag cement-based composite material, main cracks were not observed, and the top soil damage was serious. Moreover, at a confining pressure of 0.5 MPa, the main crack was observed on the diagonal of the nickel–iron slag cement-based composite, accompanied by several intricate small cracks. This part was seriously damaged, forming a crack grid with the main crack and the loading ends. At a confining pressure of 0.8 MPa, the main crack was observed at ~65° from the top of the nickel–iron slag cement-based composite, some secondary cracks were observed at the upper end of the main crack, and the width of the main crack was considerably less than those of the nickel–iron slag cement-based composite at confining pressures of 0.2 and 0.5 MPa. Moreover, the macroscopic crack penetration damage was developed in the vertical and parallel directions.

### 3.2. Influence of Sodium Sulfate Concentration on the Stress–Strain Relation of Nickel–Iron Slag Cement-Based Composites

**Figure 8:**
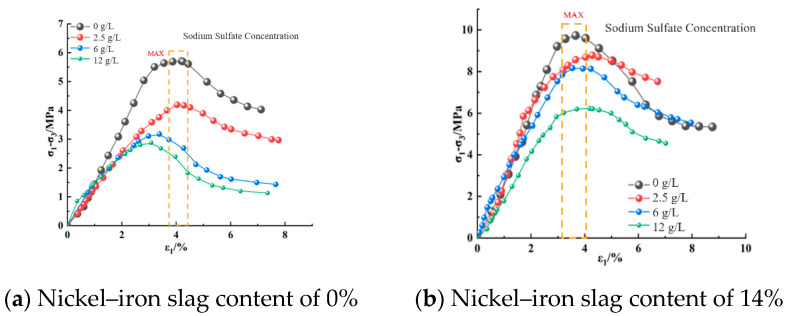
Stress–strain curve of the composites at a confining pressure of 0.2 MPa.

**Figure 9:**
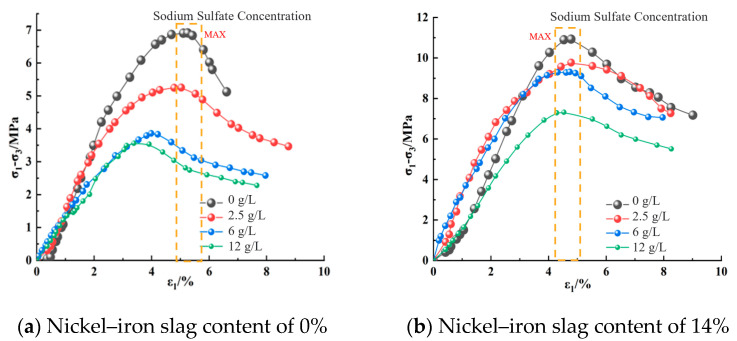
Stress–strain curve of the composites at a confining pressure of 0.5 MPa.

**Figure 10:**
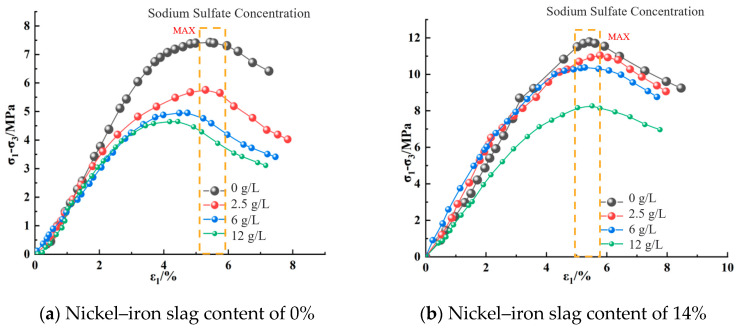
Stress–strain curve at a confining pressure of 0.8 MPa.

Figure 8, Figure 9 and Figure 10 show the effect of the sodium sulfate concentration on the stress–strain relation of cement-based composites with 0% nickel–iron slag content and 14% nickel–iron slag content at confining pressures of 0.2, 0.5, and 0.8 MPa.

**Figure 11:**
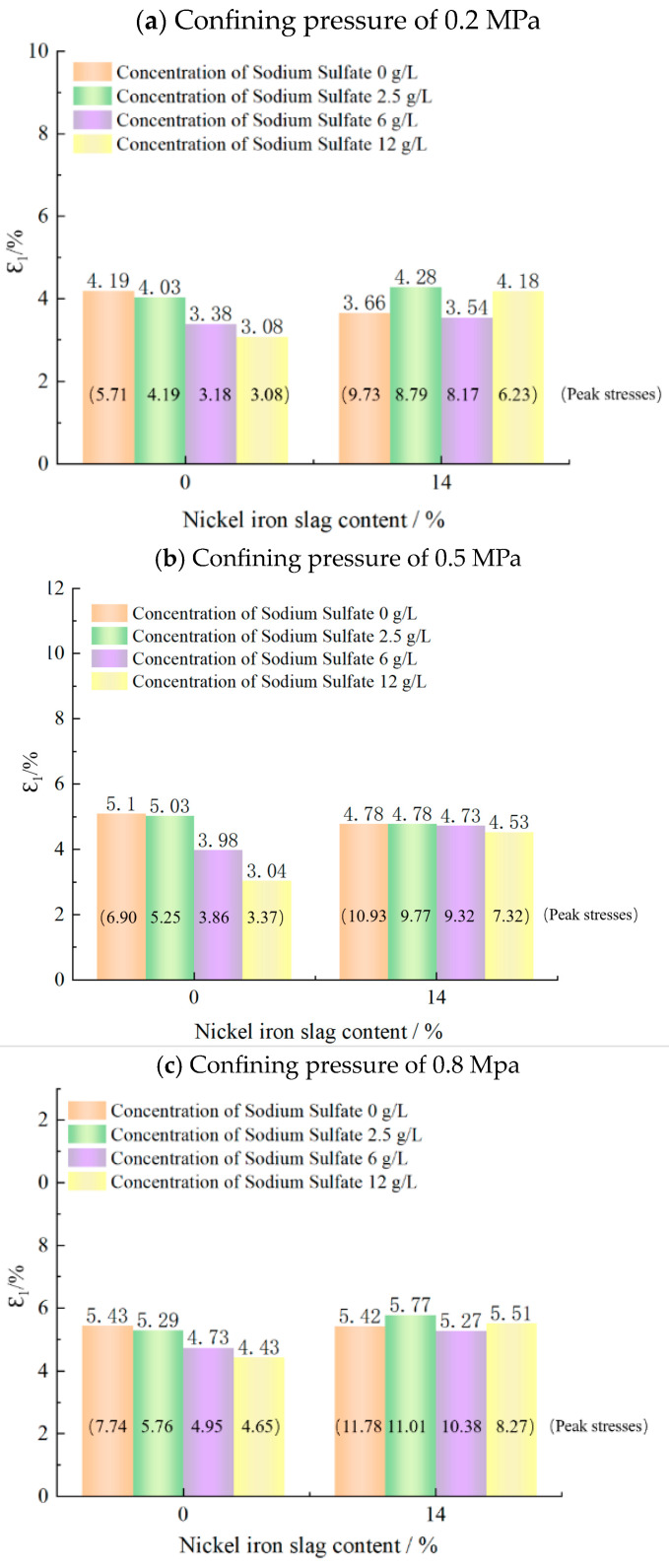
Strain values corresponding to the peak stresses at different confining pressures.

The stress–strain relation curve of nickel–iron slag cement-based composite materials reflected softening, which mainly passes through the compaction, elastic, yield, and failure stages (Figure 8, Figure 9 and Figure 10). At the same confining pressure and the same content of the nickel–iron slag, the strength of the nickel–iron slag cement-based composites at a sodium sulfate concentration of 0 g/L was considerably greater than that at sodium sulfate concentrations of 2.5, 6, and 12 g/L. Moreover, the peak stress–strain curve of the nickel–iron slag cement-based composite at a sodium sulfate concentration of 0 g/L was more obvious than that of the nickel–iron slag cement-based composite at sodium sulfate concentrations of 2.5, 6, and 12-g/L, and the peak strain was observed at a sodium sulfate concentration of 12 g/L. Sodium sulfate exhibited an erosion effect on the nickel–iron slag cement-based composite, which decreased its strength and brittleness and increased its ductility. Figure 11 shows the strain values corresponding to the peak stress values.

### 3.3. Influence of Sodium Sulfate Concentration on the Shear Strength of Nickel–Iron Slag Cement-Based Composite Materials

**Figure 12:**
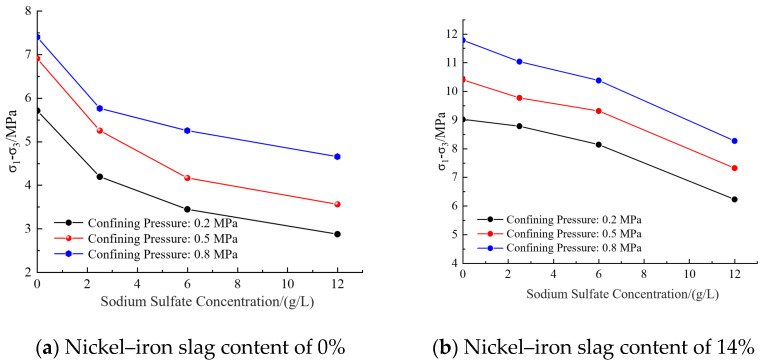
Effect of sodium sulfate concentration on shear strength.

Figure 12a shows the effect of the sodium sulfate concentration on the shear strength of cement-based composites at a nickel–iron slag content of 0%. At a confining pressure of 0.2 MPa, the shear strength values at sodium sulfate concentrations of 2.5, 6, and 12 g/L were 22.1%, 28.9%, and 37.1% less than that at a sodium sulfate concentration of 0 g/L, respectively. At a confining pressure of 0.5 MPa, the shear strength values at sodium sulfate concentrations of 2.5, 6, and 12 g/L were 23.9%, 26.5%, and 48.4% less than that at a sodium sulfate concentration of 0 g/L, respectively. At a confining pressure of 0.8 MPa, the shear strength values at sodium sulfate concentrations of 2.5, 6, and 12 g/L decreased by 26.5%, 39.7%, and 49.6%, respectively, as compared to that at a sodium sulfate concentration of 0 g/L.

Figure 12b shows the effect of the sodium sulfate concentration on the shear strength of the nickel–iron slag cement-based composite at a nickel–iron slag content of 14%. Under a confining pressure of 0.2 MPa, the shear strength values at sodium sulfate concentrations of 2.5, 6, and 12 g/L were 2.6%, 9.8%, and 15.4% less than that at a sodium sulfate concentration of 0 g/L, respectively. Moreover, under a confining pressure of 0.5 MPa, the shear strength values at sodium sulfate concentrations of 2.5, 6, and 12 g/L sodium sulfate decreased by 6%, 10.4%, and 19.6%, respectively, in comparison to that at a sodium sulfate concentration of 0 g/L. Under a confining pressure of 0.8 MPa, the shear strength values at sodium sulfate concentrations of 2.5, 6, and 12 g/L were 6.5%, 11.9%, and 20.7% less than that at a sodium sulfate concentration of 0 g/L, respectively.

### 3.4. Influence of Sodium Sulfate Concentration on the Cohesion and Internal Friction Angle of the Nickel–Iron Slag Cement-Based Composites

Under different contents of nickel–iron slag (0%, 14%), confining pressures (0.2, 0.5, and 0.8 MPa), and sodium sulfate concentrations (0, 2.5, 6, and 12 g/L), changes in the shear strength of the nickel–iron slag cement-based composite materials with normal stress were investigated by the Coulomb law. The shear strength is expressed as follows:

(1) τ=σtan⁡φ+c

In Equation (1), *τ* represents the shear strength of the nickel–iron slag cement-based composite (MPa), *σ* represents the normal stress, *φ* represents the internal friction angle (°), and *c* represents cohesion (MPa).

According to Equation (1) and the regression curve, *c* and *φ* can be obtained, where the intercept between the regression curve and the vertical coordinate is *c* and the angle between the regression curve and horizontal coordinate is *φ*.

**Figure 13:**
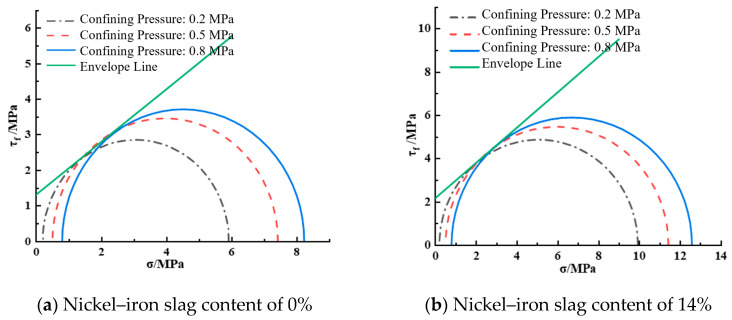
Shear strength envelope line at a sodium sulfate concentration of 0 g/L.

Figure 13 shows the envelope diagram of the shear strength of the nickel–iron slag cement-based composite at a sodium sulfate concentration of 0 g/L. At a nickel–iron slag content of 0%, the cohesion was 1.311 MPa and the internal friction angle was 36.56° according to Equation (1). At a nickel–iron slag content of 14%, the cohesion was 2.16 MPa and the internal friction angle was 40.79° according to Equation (1).

**Figure 14:**
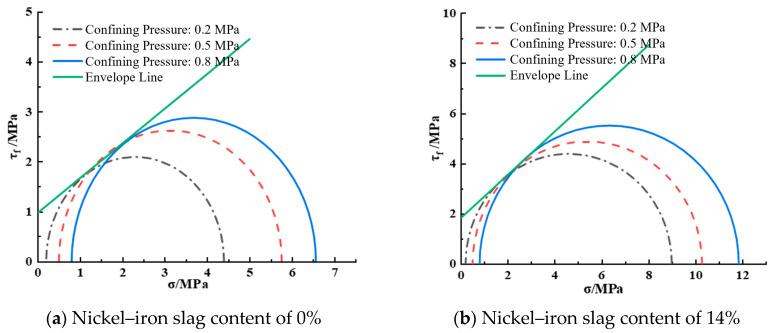
Shear strength envelope line at a sodium sulfate concentration of 2.5 g/L.

Figure 14 shows the shear strength envelope diagram of the nickel–iron slag cement-based composite at a sodium sulfate concentration of 2.5 g/L. When considering a nickel–iron slag content of 0%, the cohesion was 0.97 MPa and the internal friction angle was 34.89°. When considering a nickel–iron slag content of 14%, the cohesion was 1.83 MPa and the internal friction angle was 40.37°.

**Figure 15:**
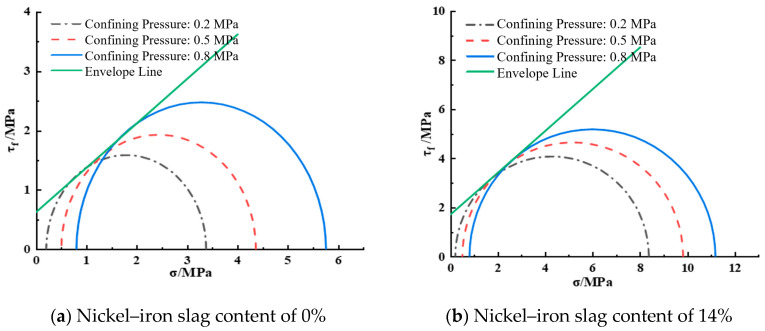
Shear strength envelope line at a sodium sulfate concentration of 6 g/L.

Figure 15 shows the shear strength envelope diagram of the nickel–iron slag cement-based composite at a sodium sulfate concentration of 6 g/L. At a nickel–iron slag content of 0%, the cohesion was 0.63 MPa and the internal friction angle was 33.08°. At a nickel–iron slag content of 14%, the cohesion was 1.72 MPa and the internal friction angle was 39.86°.

**Figure 16:**
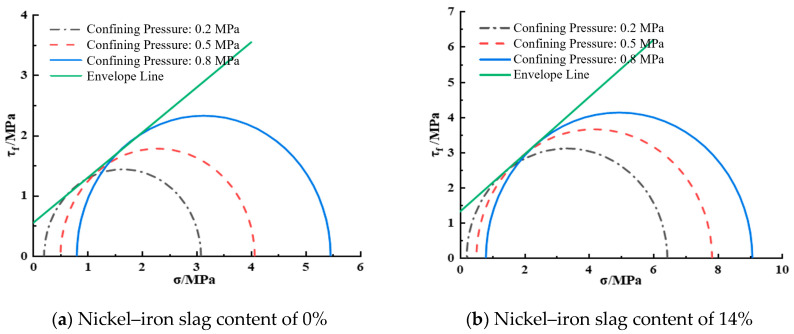
Shear strength envelope line at a sodium sulfate concentration of 12 g/L.

Figure 16 shows the shear strength envelope diagram of the nickel–iron slag cement-based composite at a sodium sulfate concentration of 12 g/L. At a nickel–iron slag content of 0%, the cohesion was 0.55 MPa and the internal friction angle was 32.61° according to the shear strength equation. At a nickel–iron slag content of 14%, the cohesion was 1.48 MPa and the internal friction angle was 39.25° according to the shear strength equation.

**Figure 17:**
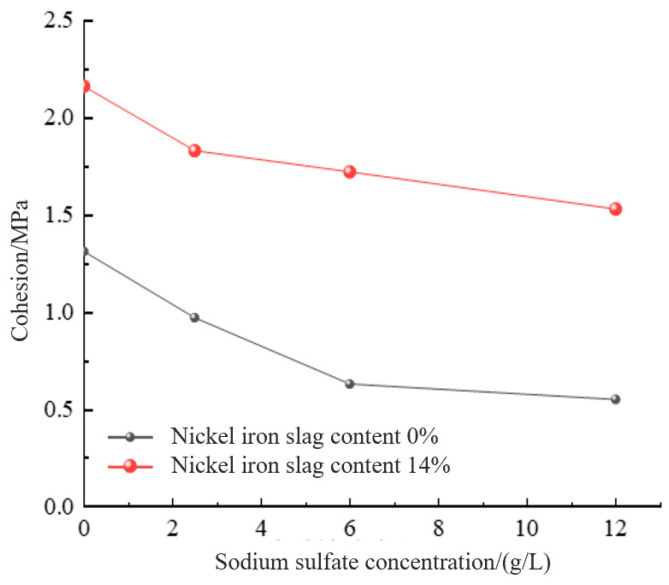
Effect of sodium sulfate concentration on cohesion.

**Figure 18:**
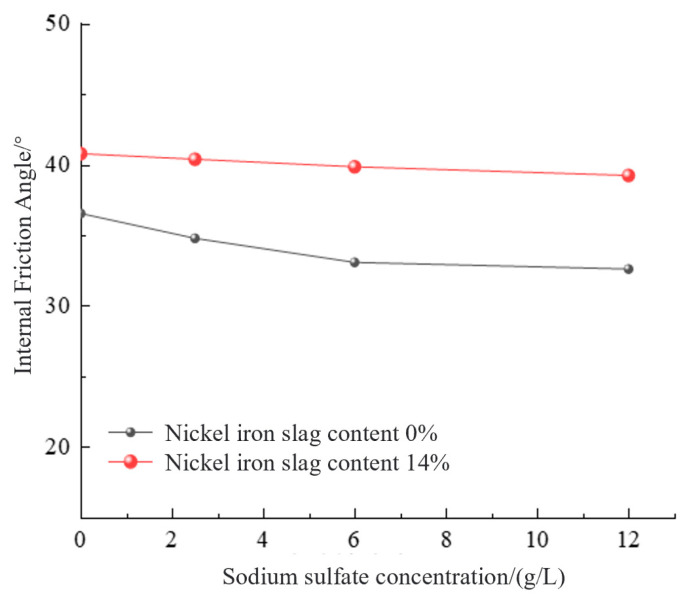
Effect of sodium sulfate concentration on the internal friction angle.

To further investigate the effect of sodium sulfate concentration on shear strength, the effects of sodium sulfate concentration on the cohesion and internal friction angle were analyzed. With the increase in the sodium sulfate concentration, the cohesion and internal friction angle decreased gradually (Figure 17 and Figure 18). The cohesion of the cement-based composite without the nickel–iron slag, at sodium sulfate concentrations of 2.5, 6, and 12 g/L, decreased by 26%, 51.9%, and 58.1%, respectively, compared with that at a sodium sulfate concentration of 0 g/L. The bonding strength values of the nickel–iron slag cement-based composite with a nickel–iron slag content of 14%, at sodium sulfate concentrations of 2.5, 6, and 12 g/L, decreased by 10.6%, 15.7%, and 19.9%, respectively, compared with that at a sodium sulfate concentration of 0 g/L. The internal friction angles of the cement-based composites without nickel–iron slag, at sodium sulfate concentrations of 2.5, 6, and 12 g/L, decreased by 1.77°, 3.48°, and 3.95°, respectively, compared with that at a sodium sulfate concentration of 0 g/L. The internal friction angle of the nickel–iron slag cement–soil composite with a nickel–iron slag content of 14%, at sodium sulfate concentrations of 2.5, 6, and 12 g/L, reduced by 0.4°, 0.93°, and 1.54°, respectively, compared with that at a sodium sulfate concentration of 0 g/L. Therefore, the effect of the optimal content of the nickel–iron slag on cohesion is considerably greater than that of the internal friction angle, and the influence of the internal friction angle continuously decreases.

### 3.5. Microscopic Mechanism of the Nickel–Iron Slag Cement-Based Composites under the Action of Sodium Sulfate

**Figure 19:**
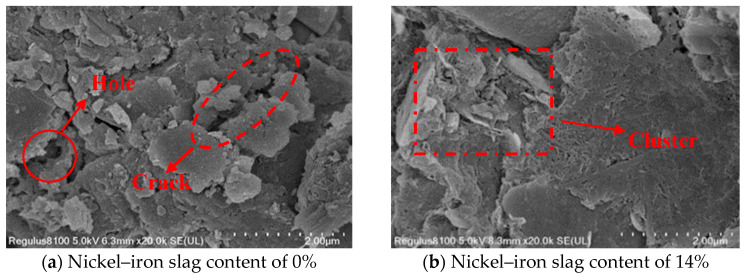
Scanning electron microscopy (SEM) image of the composite at a sodium sulfate concentration of 0 g/L at 20,000× magnification.

**Figure 20:**
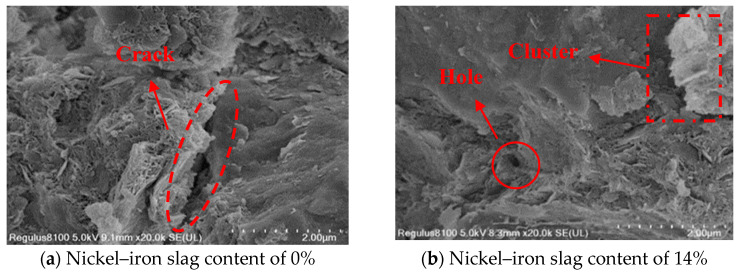
SEM image of the composite at a sodium sulfate concentration of 2.5 g/L at 20,000× magnification.

**Figure 21:**
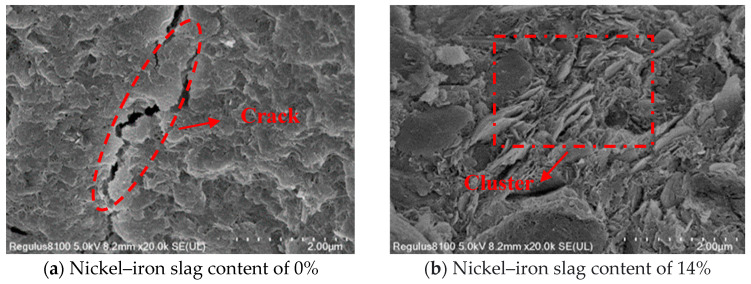
SEM image of the composite at a sodium sulfate concentration of 6 g/L at 20,000× magnification.

**Figure 22:**
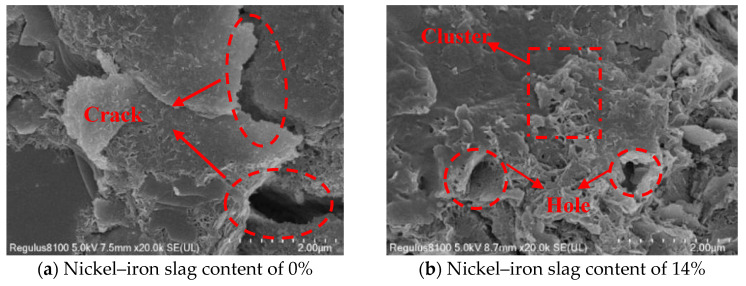
SEM image of the composite at a sodium sulfate concentration of 12 g/L at 20,000× magnification.

Ordinary Portland cement is mainly composed of tricalcium silicate, dicalcium silicate, tricalcium aluminate, and tetralcium ferroaluminate. Under the action of sodium sulfate, the change law of the influence of the nickel–iron slag content on the nickel–iron slag cement-based composite materials is mainly based on the cementation mode, compactness, particle characteristics, and microscopic arrangement of the soil’s internal structure. The damage mechanism and macroscopic characteristics of the cement-based composite materials are explained through SEM microscopic images. In the experiment, cement-based composites with a cement content of 16%, sodium sulfate concentrations of 0, 2.5, 6, and 12 g/L, and nickel–iron slag contents of 0% and 14% were selected as the research objects, and the SEM images of the microstructures of the different composites were recorded at 20,000× magnification (Figure 19, Figure 20, Figure 21 and Figure 22).

Figure 19a, Figure 20a, Figure 21a and Figure 22a show the SEM images of soil–cement at sodium sulfate concentrations of 0, 2.5, 6, and 12 g/L, respectively. Several aggregates were generated in the soil without sodium sulfate erosion, and small areas of aggregates were distributed around the soil (Figure 19a). Pores and holes were also observed, and the structure was not dense. The soil contained a small number of needle-like structures (Figure 20a). Previous studies [37] reported that needle-like structures visualized in SEM images are gypsum or calcium vanite crystals formed from the reaction of sulfate ions with cement. These needle-like structures are present in clusters, mostly in the form of short columnar strips. In the early stage, the needle-like structures can be used as fillers to fill internal pores and holes, and the structures were relatively dense. These needle-like structures continue to grow and form swells, which gradually create cracks within the soil mass. With an increase in the sodium sulfate concentration, increased amounts of gypsum or calcium stone are generated by hydration. Hence, the cracks inside the soil were deepened, and faults were observed (Figure 21a) because the swelling force generated by the gypsum or calcium stone will expand the pores inside the soil, and most of the unreacted swelling materials will be deposited in these pores. The internal structure of the cement composite soil was gradually spaced out, while additional sulfate ions continued to be added into the soil, resulting in the formation of large quantity of products with erosive properties as well as a higher expansion effect; hence, the internal structure became loose, and the soil was destroyed. Sherwood [38] demonstrated that the formation of expansive minerals ettringite and wolfrite gypsum was the main reason for the expansion of cement-improved soil.

Figure 19b, Figure 20b, Figure 21b and Figure 22b show the SEM images of the nickel–iron slag cement–soil composite at sodium sulfate concentrations of 0, 2.5, 6, and 12 g/L. The internal structure of the soil exhibited good compactness and integrity. At a low sodium sulfate concentration, the material generated by the reaction of sulfate ions with the hydration products of cement can fill the pores inside the soil. Moreover, because the nickel–iron slag was mainly composed of silica, it exhibited a strong pozzolanic activity, and the gelling material generated after the reaction with cement covered the structure’s skeleton, enhancing the compactness of the structure. With the increase in the sodium sulfate concentration, many high-concentration sulfate ions flooded into the soil, and the amount of swelling material accumulated by hydration was greater than the carrying capacity of the pores (Figure 19b and Figure 20b). Small cracks and a few holes were observed, and the internal structure was denser and more monolithic than that of plain hydraulic soil (Figure 22b).

## 4. Conclusions

(1) Under the action of sodium sulfate, the stress–strain curve of the nickel–iron slag cement-based composite reflects softening, and the shear strength of the nickel–iron slag cement-based composite gradually decreases with the increasing sodium sulfate concentration. The optimum 14% nickel–iron slag addition leads to the increase in its plasticity and decrease in its brittleness.
(2) With the increase in the sodium sulfate concentration, the cohesion and internal friction angle of the cement-based composite decrease. The decrease in the cohesion and internal friction angle of the cement-based composite derived from the nickel–iron slag is low in the case of a nickel–iron slag content of 14%. The addition of nickel–iron slag can delay the reduction in the cohesion and internal friction angle; hence, the corrosion resistance of the cement-based composite can be improved.
(3) SEM images under 20,000× magnification reveal that with the increase in the sodium sulfate concentration and under a nickel–iron slag content of 14%, no clear cracks are observed inside the cement-based composite material. Adequate synergetic reaction occurs between SiO_2_ and cement in the nickel–iron slag structure, resulting in a large number of gel granule structures inside the cement-based composite material. Thus, with increasing nickel–iron slag content, the internal pores are filled tightly, the degree of cementation is increased, the corrosion of the cement–soil composite by sulfate ions is effectively inhibited, and the corrosion resistance of the nickel–iron slag cement-based composite material is improved.

This study reveals that sodium sulfate has an erosive effect. It reduces the mechanical properties and soil erosion resistance of cement composites. However, nickel–iron slag incorporation to cement can reduce the erosion of cement–soil composites by sodium sulfate. To investigate the erosive effect of the salt solution, this experiment only considered the influence law of the number of freeze–thaw cycles of the salt solution on nickel–iron slag cement–soil composite. Hence, the influence law of different water-to-cement ratios on the mechanical properties and durability performance of nickel–iron slag cement-based composites can also be considered. EDS analysis can be performed simultaneously with SEM. In addition, further research is needed to consider the influence of sodium chlorate, acidity and alkalinity, and other complex erosive environments on the change pattern of the mechanical properties and durability performance of cement–soil composite mixed with nickel–iron slag.

## Figures and Tables

**Table 1:** Chemical composition of the nickel–iron slag.

Composition	SiO_2_	Al_2_O_3_	Na_2_O	K_2_O	Fe_2_O_3_	CaO	TiO_2_
Proportion (%)	72.72	16.76	5.25	2.97	1.85	0.29	0.16

**Table 2:** Results of the physical behaviors of silty clay.

Natural Moisture Content, w (%)	Natural Weight, γ (kN/m^−3^)	Natural Soil Density, ρ (g/cm^−3^)	Liquid Limit, wL (%)	Plastic Limit, wP (%)	Liquidity Index, IP	Plasticity Index, IL
27.0	19.5	1.94	33.3	18.5	0.54	14.6

**Table 3:** Physical and mechanical indexes of the cement.

Testing Content	Normal Consistency (%)	Initial Setting Time (min)	Final Setting Time (min)	Loss on Ignition (%)	Compressive Strength (MPa)		Tensile Strength (MPa)	
					3 Days	28 Days	3 Days	28 Days
Measured value	29.3	194	392	1.04	23.7	47.1	5.6	7.1

**Table 4:** Chemical composition of sodium sulfate.

Composition	Na_2_SO_4_	Cl	Ca	K	SO_4_	PO_4_	Water-Insoluble Substance
Content (%)	≥99.0	≤0.001	≤0.002	≤0.02	≤0.002	≤0.001	≤0.005

## Data Availability

The data used to support the findings of this study are available from the corresponding author upon request.
